# Critical appraisal and future directions for peripheral nerve blocks for hip fracture surgeries: a bibliometric and visual analysis: correspondence

**DOI:** 10.1097/JS9.0000000000002989

**Published:** 2025-07-07

**Authors:** Aikebaierjiang Aisaiti, Han Ran, Aikeremujiang Muheremu

**Affiliations:** a Orthopedic Research Center, Sixth Affiliated Hospital of Xinjiang Medical University, Urumqi, Xinjiang, China; b Department of Orthopedics, Sixth Affiliated Hospital of Xinjiang Medical University, Urumqi, Xinjiang, China; c Department of Pediatric Orthopedics, Sixth Affiliated Hospital of Xinjiang Medical University, Urumqi, Xinjiang, China

*Dear Editor,*

The article is compliant with the TITAN Guidelines 2025—governing declaration and use of AI: https://doi.org/10.70389/PJS.100082^[1]^.

We read with great interest the article by Zhang *et al* titled “Peripheral Nerve Blocks (PNB) for Hip Fracture Surgeries: A Bibliometric and Visual Analysis”^[2]^. This comprehensive analysis provides valuable insights into the evolving landscape of PNB research for hip fracture management. Its most significant contribution lies in systematic mapping of global research trends, demonstrating a remarkable 31.81% annual growth in PNB-related publications. The identification of emerging techniques like the PENG block and modified fascia iliaca approaches, along with their associated benefits in postoperative recovery, provides crucial guidance for clinicians.

While we commend the authors for their meticulous bibliometric approach, we wish to highlight several critical aspects that could further strengthen both the interpretation of current findings and future research directions in this important field.

First, the study identifies multiple PNB approaches but does not sufficiently address how clinicians should choose between them. For instance, while the PENG block shows promise for preserving motor function^[3]^, how does its analgesic efficacy compare to traditional fascia iliaca blocks in different surgical scenarios? A more nuanced discussion of indications and contraindications for each technique would significantly enhance clinical utility.

Second, the barriers to real-world implementation of PNB, particularly in resource-limited settings, warrant deeper exploration. Beyond mentioning ultrasound costs, the discussion could address training requirements and learning curves for different techniques; Solutions for settings without advanced imaging equipment; Strategies for implementing PNBs in emergency departments where hip fractures often first present.

Third, the focus on short-term analgesic outcomes, while important, overlooks critical long-term considerations such as the impact of PNB on functional recovery and mobility at 3–6 months post-operation, potential effects on chronic pain development as well as its influence on rehabilitation timelines and success.

Forth, the study’s exclusive focus on English-language literature may introduce selection bias^[4]^, particularly given the global burden of hip fractures. Many high-quality studies published in other languages such as Chinese, Japanese and Scandinavian languages may contain valuable data that could alter the observed trends. This limitation should be more prominently acknowledged and its potential impact discussed.

Additionally, while the bibliometric approach effectively maps publication trends, it cannot substitute for systematic reviews of clinical outcomes^[5]^. The authors might consider emphasizing this distinction more clearly to prevent potential misinterpretation of the findings as clinical recommendations.

Building on this important work, we suggest several key areas for future investigation: (1) Development of core outcome sets for PNB studies in hip fractures would enable more meaningful comparisons across techniques and studies. These should include both clinician-reported and patient-reported outcomes. (2) Given the economic burden of hip fractures, rigorous health economic evaluations comparing different PNB approaches to traditional analgesia are urgently needed. (3) Particular attention should be paid to patients with cognitive impairment, the very elderly (>85 years), and those with multiple comorbidities. (4) Research examining real-world adoption barriers and facilitators could bridge the gap between evidence and practice.

In sum, Zhang *et al* have provided an invaluable foundation for understanding the evolution of PNB research in hip fracture management. By addressing these additional considerations in future work, the field can move toward more evidence-based, patient-centered approaches that optimize both short-term recovery and long-term outcomes. We encourage the authors to consider these perspectives as they continue their important work in this area.

## Data Availability

Not applicable.
